# Efficacy of an RNA-based multigene assay with core needle biopsy samples for risk evaluation in hormone-positive early breast cancer

**DOI:** 10.1186/s12885-019-5608-2

**Published:** 2019-04-25

**Authors:** Jeeyeon Lee, Eun Hye Lee, Ho Yong Park, Wan Wook Kim, Ryu Kyung Lee, Yee Soo Chae, Soo Jung Lee, Jee-Eun Kim, Byeong-il Kang, Jee Young Park, Ji-Young Park, Jin Hyang Jung

**Affiliations:** 1 Department of Surgery, School of Medicine, Kyungpook National University, Kyungpook National University Hospital, Daegu, Republic of Korea; 2 Department of Pathology, School of Medicine, Kyungpook National University, Kyungpook National University Hospital, Daegu, Republic of Korea; 3 Department of Hemato-Oncology, School of Medicine, Kyungpook National University, Kyungpook National University Hospital, Daegu, Republic of Korea; 4 R&D Center, Gencurix Inc., Seoul, Republic of Korea

**Keywords:** Breast, Carcinoma, Gene, Biopsy

## Abstract

**Background:**

Gene expression profiling provides key information for prognosis of breast cancer to establish treatment strategy. However, the genetic assessment should be available before induction of treatment to be useful for clinical practice. To evaluate the reliability of using needle biopsy samples for gene assays, we compared gene-expression profiling results between core needle biopsy (CNB) samples and surgical specimens in breast cancer.

**Methods:**

Thirty-one paired, formalin-fixed, paraffin-embedded CNB and surgical specimen samples were selected from patients with hormone receptor-positive breast cancer. Total RNA was extracted from the samples and the risk classifications based on GenesWell BCT scores were compared.

**Results:**

The BCT scores correlated between CNB samples and surgical specimens of hormone receptor-positive breast cancer (Pearson *r* = 0.66). The overall concordance rate of risk classification (high/low risk) was 83.9%. However, when the breast cancer does not contain intratumoral microcalcification, the concordance rate increased as 92.0%. And, when the breast cancer formed a solitary nodule (non-multifocal), the concordance rate increased up to 95.8%.

**Conclusion:**

Risk classification using the GenesWell BCT multigene kit with CNB samples could be considered reliable, when the breast cancer is a solitary nodule without intratumoral microcalcification. Such genetic profiling results should be helpful for establishing a treatment plan for hormone receptor-positive breast cancer before treatment induction.

## Background

Breast cancer is a major health problem in women worldwide. It is the most frequent malignancy in women, with nearly 1.4 million new cases and 0.5 million breast cancer-related deaths [1]. Moreover, the incidence of breast cancer has been increasing gradually, in South Korea, with more than 20% increase in 2012 compared to 2008 [2]. Among newly diagnosed breast cancer patients, about 80% of have hormone-positive breast cancer with early-staged tumors, and can therefore expect a low risk of recurrence, which means that they may not need adjuvant chemotherapy or radiotherapy [3]. Commercialized multigene kits, such as Oncotype DX®, EndoPredict®, Mammaprint®, have been approved to estimate the risk of local relapse or distant metastasis [4–6].

Gene expression profiling, which can be used to guide treatment, has emphasized the importance of tumor proliferation as a prognostic factor for hormone receptor-positive breast cancer [7, 8]. Numerous studies have examined multigene signatures in order to investigate whether the major genes related to tumor proliferation and apoptosis are associated with prognosis [9–13]. This work is ongoing; indeed, the companies which produce commercial multigene kits are still searching for additional key factors with greater relevance to breast cancer. Meanwhile, a 9-gene test, GenesWell BCT (Gencurix Inc., Seoul, South Korea), developed domestically in South Korea, has been granted limited approval for estimating risk of distant metastasis within the first 10 years of early, hormone receptor-positive, HER2-negative breast cancer with a prognostic risk score [14]. The present study focused on this kit.

To be useful for clinical practice, gene expression profiling should be available before induction of treatment [15]. However, since multigene kits are usually developed with surgical specimens, and commercial multigene tests have not been extensively studied with needle biopsy samples, they are not comprehensively validated for such use [16–18]. Even if the core needle biopsy (CNB) shows highly accurate results in determining estrogen receptor (ER), progesterone receptor (PR), and HER2 status [19–21], there is a degree of uncertainty in predicting prognosis using multigene test kits with needle biopsy samples prior to initial treatment.

In the present study, we compared the degree of conformity between gene-expression signatures from CNB and surgical specimen samples from the same patient. We used a newly developed multigene test kit (GenesWell BCT) to evaluate the reliability of gene-expression profiling from needle biopsy for determining treatment plan in early, hormone-positive breast cancer prior to surgery.

## Methods

### Ethical approval

Informed consent was obtained from all patients and the protocol used in this study was approved by the Institutional Review Board (IRB) Committee of Kyungpook National University Chilgok Hospital, Daegu, Republic of Korea (2018-06-014). And the specific inclusion and exclusion criteria were defined in the approved IRB protocol. The informed consents were obtained by written documents from all patients.

### Study population and tissue preparation

Thirty-six consecutive female patients with early, hormone-positive breast cancer who underwent curative surgery were initially included. However, five of these patients were excluded from our final analysis because of chemotherapy before surgery (*n* = 1), recurrent breast cancer (*n* = 1), inadequate tumor content in specimens (*n* = 1), or negative immunohistochemical expression of ER and PR at confirmatory stain test (*n* = 2).

A total of 31 formalin-fixed, paraffin-embedded (FFPE) samples paired with core needle biopsies and surgical specimens were evaluated. Clinical information used included patients’ age, body mass index, clinical and pathologic tumor size, nodal status, pathologic stage, a follow-up period, oncologic outcomes and specific clinical features of breast cancer.

Three or four cores of CNB samples were taken with 16G-core needles for breast mass at diagnosis and surgical specimens were obtained from conventional breast cancer surgery. Both CNB samples and surgical specimens were treated as conventional FFPE tissue sections, and three consecutive 10 μm-curl type sections were obtained from the FFPE tissue. Tumor volume (%) was estimated by screening of representative H & E slides and calculated as the ratio between tumor volume and total parenchyma volume, with fat tissues excepted. Each specimen was selected as representative of its tumor block after review by pathologists. All the breast cancers were diagnosed as invasive ductal carcinoma both in CNB and surgical specimen samples.

### Histopathologic examination

Molecular subtyping was based on the immunohistochemical expression of the ER, PR, and HER2/neu gene amplification by silver in situ hybridization (SISH). To define positive expression of ER/PR/HER2, we used the criteria from the ASCO/CAP 2016 guidelines for histopathologic examination.

### Total RNA extraction

Three consecutive FFPE tissue sections with 10 μm thickness were prepared for each CNB and the corresponding surgical specimens. We used a fully automated isolation method for total RNA extraction and DNase I treatment from all FFPE tissue sections. This method was based on the manufacturer’s manual, and used a tissue preparation system (Siemens Healthcare Diagnostics, Munich, Germany) and VERSANT tissue preparation reagents kit (Siemens Healthcare Diagnostics, Munich, Germany). For each FFPE section, 100 μL of extracted total RNA was preserved at − 80 °C.

### GenesWell BCT assay and BCT score

The GenesWell BCT assay was carried out to determine the expression levels (Cp values) of six prognostic genes (*UBE2C, TOP2A, RRM2, FOXM1, MKI67,* and *BTN3A2*) and three reference genes (*CTBP1, CUL1,* and *UBQLN1*). Gene expression levels were measured by quantitative one-step reverse transcription PCR using a QuantiFast Multiplex RT-PCR + R Kit (Qiagen, Hilden, Germany) on a LightCycler 480 II (Roche Diagnostics, Basel, Switzerland).

The BCT score was calculated from the relative expression levels (ΔCp values) of the six prognostic genes normalized with the three reference genes, in conjunction with clinical information (pathologic tumor size and nodal stage). In the case of core needle biopsies, clinical tumor size and nodal stage were used as clinical information. Each specimen was classified as low or high risk of distant relapse based on a pre-defined cutoff BCT score of 4.0, as suggested in a previous study [22]. The concordance between risk classification in surgical and CNB specimens was identified and the correlation of BCT score between these two groups was analyzed.

### Statistical analysis

We used Pearson’s correlation to compare concordance between groups. Significance was inferred where *P* < 0.05. All average values are given as mean ± SD.

## Results

### Patients’ characteristics and clinical outcomes

Among thirty-six paired samples with FFPE tumor sections and CNB samples, thirty-one eligible, paired samples were used in the current study. The mean age of the 31 patients was 58.7 ± 8.3 years and their mean body mass index (BMI) was 24.6 ± 3.3. The mean clinical and pathologic tumor sizes were 1.4 ± 0.7 cm and 1.5 ± 0.5 cm, respectively. Sever cases (22.6%) showed multifocality in preoperative imaging findings and final pathologic report. Pathologic tumor stages were as follows: IA (*n* = 23, 74.2%); IB (*n* = 1, 3.2%); IIA (*n* = 7, 22.6%). There were two cases (6.5%) of locoregional recurrence during 40.2 ± 28.6 months of mean follow-up period (Table 1).

**Table 1:** Characteristics of thirty-one breast cancer cases for which multigene tests were performed on both core needle biopsy samples and surgical specimens

Variables	*n* = 31
Mean age (years, ±SD)	58.7 ± 8.3
Mean body mass index (kg/m^2^, ±SD)	24.6 ± 3.3
Mean clinical tumor size (cm, ±SD)	1.4 ± 0.7
Mean pathological tumor size (cm, ±SD)	1.5 ± 0.5
Cases of multifocality (n, %)	7 (22.6)
Containing microcalcification (n, %)	6 (19.4)
Pathologic tumor stage (n, %)	
IA	23 (74.2)
IB	1 (3.2)
IIA	7 (22.6)
Mean follow-up period (months, ±SD)	40.2 ± 28.6
Locoregional recurrence (n, %)	2 (6.5)

### Comparison of the tumor volume and BCT score

We expressed tumor volume as the percentage of total parenchymal tissue volume occupied by tumor. Mean tumor volume (%) in CNB and surgical specimen samples was 60.1 ± 21.7 and 39.8 ± 16.7, respectively. There were four cases of pathologic N1 stage, for which the clinical stage was N0 (Table 2). And the BCT scores were compared between CNB samples and surgical specimens.

**Table 2:** Clinical and pathological tumor size, volume, and nodal stage of thirty-one paired samples

No.	Age	Core needle biopsy			Surgical specimen			Clinical characteristics of breast cancer
		Tumor volume (%)	Clinical tumor size (cm)	cN stage	Tumor volume (%)	Pathologic tumor size (cm)	pN stage	
#1	50’s	70	1.1	0	20	1.3	0	Solitary nodule
#2	60’s	98	2.8	0	50	2.8	0	Solitary nodule
#4	50’s	60	3.2	0	35	3.2	0	Multifocal/multicentric nodules, Containing microcalcifications
#5	60’s	30	2.5	0	60	2.2	0	Multifocal/multicentric nodules, Containing microcalcifications
#7	50’s	70	1.4	0	30	1.6	0	Solitary nodule
#8	40’s	50	1.5	0	20	1.1	0	Solitary nodule
#9	40’s	80	1.5	0	25	1.6	1	Solitary nodule
#10	40’s	80	1.0	0	40	1.5	0	Solitary nodule
#11	60’s	40	2.0	0	50	1.8	0	Solitary nodule
#13	70’s	90	3.0	0	30	2.2	0	Solitary nodule
#15	60’s	70	1.8	0	30	1.9	0	Solitary nodule
#16	40’s	20	1.2	0	50	1.5	0	Multifocal/multicentric nodules, Containing microcalcifications
#17	50’s	15	1.1	0	20	0.7	0	Solitary nodule
#19	60’s	60	1.9	0	40	1.4	0	Solitary nodule
#20	60’s	40	0.3	0	40	1.3	0	Solitary nodule
#21	50’s	70	0.9	0	60	1.2	0	Solitary nodule
#22	60’s	60	1.2	0	40	1.4	0	Solitary nodule
#23	60’s	80	0.9	0	60	1.1	1	Multifocal/multicentric nodules, Containing microcalcifications
#24	60’s	50	1.8	0	80	1.5	0	Solitary nodule
#25	50’s	60	1	0	10	1.4	1	Solitary nodule
#26	50’s	60	2	0	60	1.2	0	Multifocal/multicentric nodules
#27	60’s	80	0.9	0	60	1.1	0	Solitary nodule
#28	60’s	40	0.5	0	30	0.8	0	Solitary nodule
#29	50’s	80	1.1	0	10	1.4	0	Solitary nodule
#30	60’s	40	1.6	0	40	1.8	0	Multifocal/multicentric nodules, Containing microcalcifications
#31	70’s	50	1	0	50	0.9	0	Solitary nodule
#32	70’s	60	1.25	0	50	1.6	1	Solitary nodule
#33	60’s	60	1.5	0	40	1.3	0	Solitary nodule
#34	50’s	20	1	0	20	0.8	0	Solitary nodule
#35	50’s	70	0.7	0	50	1.1	0	Solitary nodule
#36	50’s	80	0.8	0	40	1.1	0	Multifocal/multicentric nodules, Containing microcalcifications

### Correlation of gene expression in paired samples

**Fig. 1:**
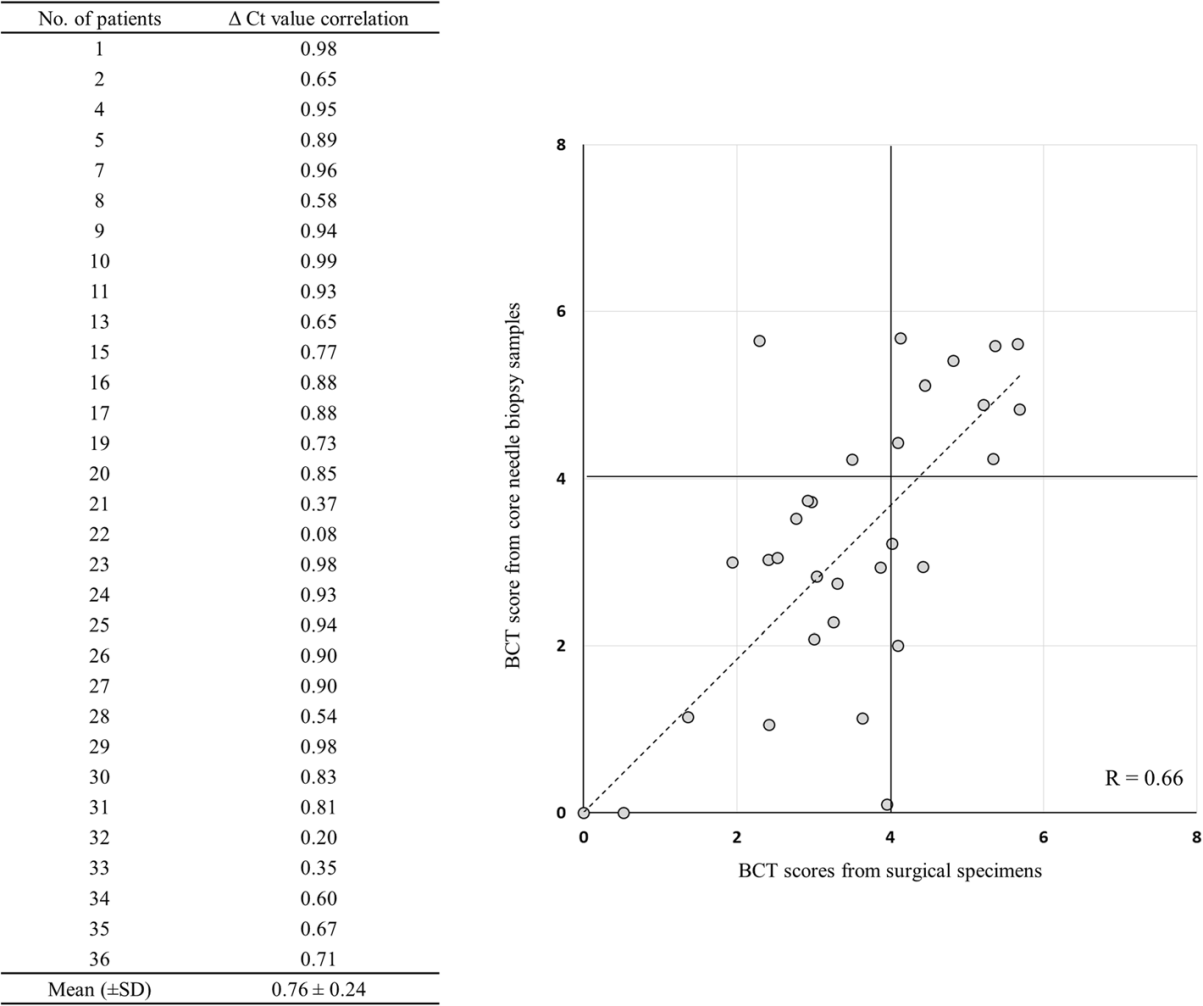
Correlations of gene expression (ΔCt value) and BCT score between paired core biopsy samples and surgical specimens in each patient. Mean correlation value was 0.76 ± 0.24 and four cases showed different risk classification between two samples

**Fig. 2:**
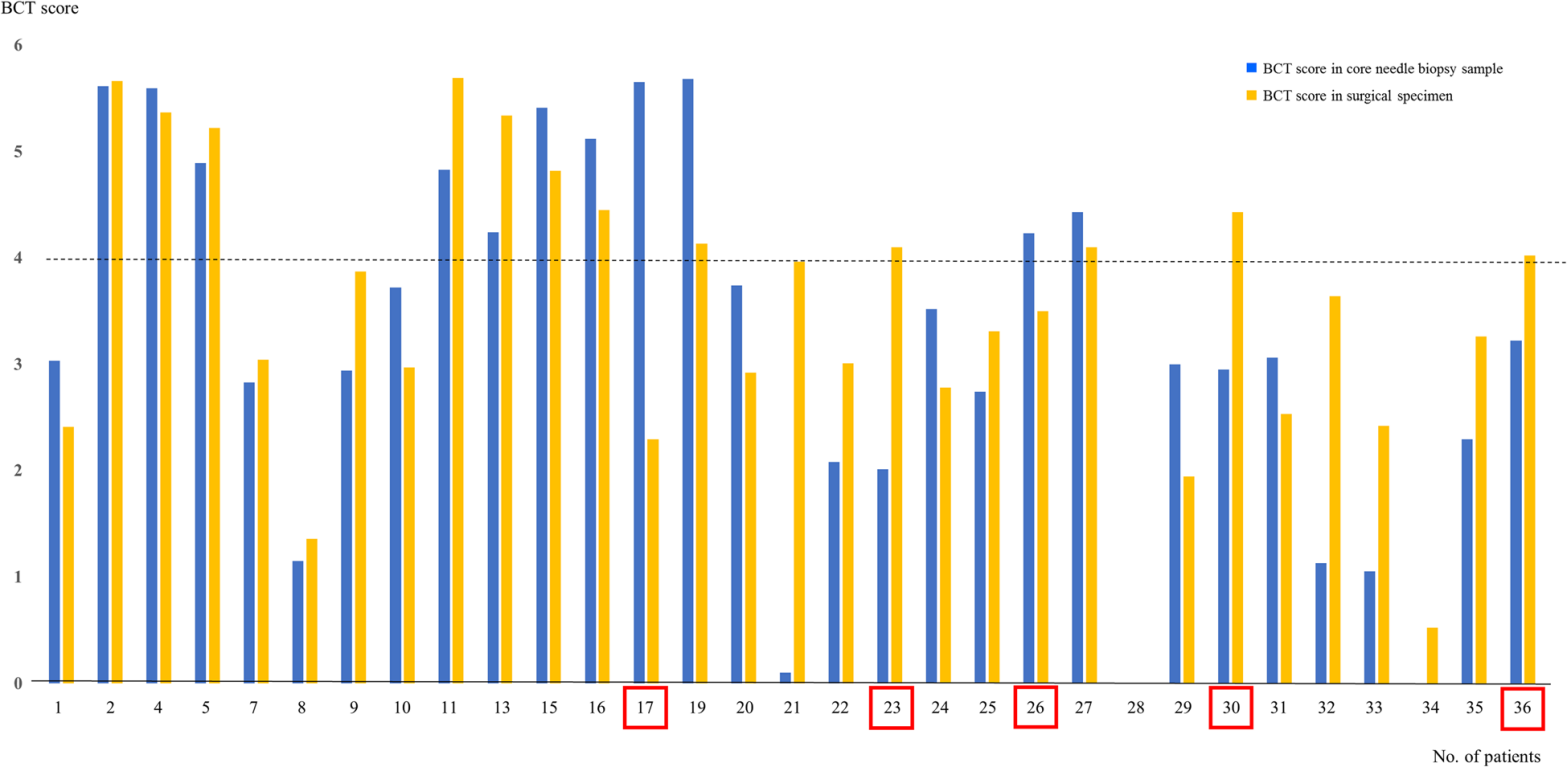
Correlation of absolute BCT scores between the paired core needle biopsy and surgical specimen samples. Based on a threshold BCT score of 4, the risk classification of five patients (no. 17, 23, 26, 30, 36) showed discordance between needle biopsy samples and surgical specimens

Gene expression (ΔCt) values in CNB samples and surgical specimens were correlated, with Pearson coefficients ranging from 0.20 to 0.99. The mean overall ΔCt value was 0.76 ± 0.24 (Fig. 1). Five case (#17, #23, #26, #30, #36) showed different risk classification between CNB and surgical samples (Fig. 2). The overall correlation rate of risk classification between CNB samples and surgical specimens was 83.9%. However, the concordance rate was higher as 92.0%, when the breast cancer was non-microcalcified lesion and 95.8%, when the breast cancer formed a solitary nodule (Table 3).

**Table 3:** Concordance of risk classification between core needle biopsy samples and surgical specimens

	Core needle biopsy sample (n, %)	Surgical specimen (n, %)		Total	Concordance rate (%)
		Low risk	High risk		
All breast cancers	Low risk	17 (54.8)	3 (9.7)	20 (64.5)	83.9
	High risk	2 (6.5)	9 (29.0)	11 (35.5)	
	Total	19 (61.3)	12 (38.7)	31 (100.0)	
Non-calcified breast cancers	Low risk	17 (68.0)	1 (4.0)	18 (72.0)	92.0
	High risk	1 (4.0)	6 (24.0)	7 (28.0)	
	Total	18 (72.0)	7 (28.0)	25 (100.0)	
Solitary (Non-multifocal) breast cancers	Low risk	17 (70.8)	0	17 (70.8)	95.8
	High risk	1 (4.2)	6 (25.0)	7 (29.2)	
	Total	18 (75.0)	6 (25.0)	24 (100.0)	

## Discussion

Although the incidence of breast cancer is increasing, its mortality rate is declining as a result of more widespread screening allowing earlier diagnosis, and advances in treatment [23, 24]. However, the higher long-term survival rate of breast cancer may increase the probability of locoregional recurrence or distant metastasis. The treatment options for breast cancer include not only surgery but also other adjuvant treatments, including invasive treatment such as chemotherapy or radiotherapy, and less invasive treatment such as hormone or target therapy. Although the invasive treatments are unnecessary for several subgroups of breast cancer which show better prognosis, it is hard to distinguish those subgroups with conventional diagnostic methods [25, 26].

Genomic profiling technologies have become an effective diagnostic method to predict clinical course of breast cancer and are helpful for making decisions relating to the tailoring of its treatment cancer [15, 21, 22, 27]. Based on multigene assays, patients with better prognosis may avoid invasive treatments such as chemotherapy or radiotherapy and their associated side effects. Several commercialized multigene predictors aimed at providing genetic information to enhance prognostic medical decisions in breast cancer have been developed recently cancer [28, 29]. One example is the microarray-based 70-gene assay, MammaPrint®, launched in 2002 by the Netherlands Cancer Institute in Amsterdam. Another is the a 21-gene multiplex prognostic and predictive RT-PCR assay onco*type* DX™, which was introduced in 2004 with the Trial Assigning Individualized Options for Treatment (TAILORx) sponsored by the National Cancer Institute. Later, in 2011, the first RNA-based prognostic test, EndoPredict®, was introduced to predict the distant recurrence of ER-positive, HER2-negative breast cancer [4, 28]. These commercialized multigene assay kits were developed using FFPE or freshly frozen tissue obtained from surgery. Therefore, their development was limited by a lack of genetic information obtained prior to treatment. To overcome this limitation, studies have been conducted to confirm a satisfactory level of correlation between results obtained from needle biopsy tissue and surgical specimens [15, 19–21].

Adding further complexity, previous gene expression profiling studies have shown discordant results between different ethnicities [30–32]. GenesWell BCT was developed using six prognostic genes including five proliferative genes (*UBE2C*, *TOP2A*, *RRM2*, *FOXM1*, and *MKI67*), one gene involved in the immune system (BTN3A2) and three reference genes (*CTBP1*, *CUL1*, *UBQLN1*). These genes were selected from public microarray gene expression data, and the reliability of the BCT score produced by GenesWell BCT was validated in an independent cohort of Koreans [22].

Because this product was developed especially for ER+, HER2- breast cancer patients, who show little benefit from chemotherapy, chemotherapy can be omitted for this group when the result indicates low risk. However, the genetic information should be obtained before treatment is started.

Recently, most breast cancer is detected by screening image and diagnosed at an early stage [33, 34]. To confirm diagnosis, ultrasound-guided needle biopsy is more popular than excision. Usually three or four cores are obtained using a needle biopsy kit and tumor characteristics are analyzed with immunohistochemical staining as well as examined visually for presence of cancer cells. However, breast cancer is known to be particularly heterogeneous within the tumor, and results can differ between operators. Furthermore, 10–20% of immunohistochemical staining results disagree between needle biopsy samples and surgical specimens in assessment of molecular subtype [17, 35]. Therefore, when the breast cancer is multifocal lesion or containing heterogenous materials, including microcalcifications, the tissue concordance would be decreased between needle biopsy samples and surgical specimens.

In the present study, we investigated whether the GenesWell BCT multigene test is suitable for CNB samples in addition to surgical specimens. The samples were obtained from areas in which at least 30% of total tissue volume was tumorous, if possible. The overall concordance rate that we observed between CNB and surgical specimens was 83.9%, which was similar or lower than other commercialized multigene kits. However, when the breast tumor showed an imaging finding of solitary nodule without microcalcification, the concordance rates were higher as 92.0 and 95.8%, respectively.

The current study aimed at validating the use of the GenesWell BCT kit to improve prognostic prediction in hormone-positive breast cancer, in order to establish treatment plans prior to initial treatment. With this aim in mind, two limitations should be noted. First, the small number of paired samples cautions strong conclusions, in particular inference to other subtypes of breast cancer. Even if we conducted the study with thirty-one paired samples of hormone positive breast cancer, it is still a small study. Therefore, the further study would be necessary to validate the results and provide the stronger evidences with reproduction of results. However, it is notable that in our thirty-one paired samples of hormone-positive breast cancer, we observed a very high concordance rate between needle biopsy samples and surgical specimens when the breast cancer is not multifocal, without containing microcalcifications. The second limitation relates to the oncologic conditions of gene profiling using GenesWell BCT. Although the actual BCT scores were developed with pathologic tumor size and nodal status, in the present study we used clinical tumor size and nodal status for obtaining BCT scores from biopsy samples. However, based on preoperative breast magnetic resonance (MR) images showing quite a high concordance rate between clinical and pathologic tumor size in early breast cancer [36, 37], it is reasonable to apply clinical tumor size for assessing BCT score before treatment. Because we also evaluated tumor size with preoperative breast MR, the clinical and pathologic staging we recorded were identical in most cases. Although there was one case in which sentinel lymph node biopsy was omitted in order to shorten the operative time because of the patient’s severe underlying disease, she has not experienced locoregional recurrence or distant metastasis during the follow-up period. Therefore, the authors regarded this case as clinically and pathologically N0 stage.

## Conclusion

The correlations of risk classification obtained from the GenesWell BCT multigene kit between CNB samples and surgical specimens in hormone positive breast cancer was relatively low, but not much inferior to other proven commercialized genetic profiling products. However, the multigene tests were performed in cases of solitary breast cancer without microcalcification, the concordance rate was much higher. This data supports the possibility of using the risk scores obtained to help establish a treatment plan before starting initial breast cancer treatment, especially for a solitary nodule of breast cancer without microcalcifications.

## Abbreviations

BCT: Breast cancer test
BMI: Body mass index
CNB: Core needle biopsy
ER: Estrogen receptor
FFPE: Formalin-fixed, paraffin-embedded
MR: Magnetic resonance
PR: Progesterone receptor
SISH: Silver in situ hybridization
